# A New Faecal Immunochemical Test Buffer With Improved Haemoglobin Stability Under Varying Temperature and Storage Times

**DOI:** 10.1002/jcla.70317

**Published:** 2026-07-26

**Authors:** Rafha Rafeeu, Geraldine Laven‐Law, Jean M. Winter, Karen Wang, Rachel Parker, Graeme P. Young, Erin L. Symonds

**Affiliations:** ^1^ Flinders Health and Medical Research Institute, College of Medicine and Public Health Flinders University of South Australia Bedford Park South Australia Australia; ^2^ South Australian ImmunoGENomics Cancer Institute, Faculty of Health and Medical Sciences Adelaide University Adelaide South Australia Australia; ^3^ Department of Medicine Flinders University Bedford Park South Australia Australia; ^4^ Gastroenterology Department Flinders Medical Centre Bedford Park South Australia Australia

**Keywords:** colorectal cancer screening, diagnostic test, Faecal immunochemical test, faecal occult blood test, haemoglobin stability, temperature exposure

## Abstract

**Background:**

Measurement of faecal haemoglobin (f‐Hb) using the faecal immunochemical test (FIT) in population screening for colorectal cancer (CRC) has reduced cancer‐related incidence and mortality. However, temperature‐induced f‐Hb degradation can decrease FIT accuracy. This study assessed the f‐Hb preservation capacity of a new FIT Hb‐stabilising buffer at different temperatures and durations.

**Methods:**

Fresh whole faecal samples from individuals with active colorectal bleeding were used for collection of samples using the OC‐Sensor FIT (EIKEN CHEMICAL CO. LTD, Japan) containing either the standard (SOC3) or new formulation (SOC4) buffer. Pooled faeces‐buffer mixtures (*n* = 29) were divided into aliquots for incubation at 4°C, 23°C, 35°C, 45°C, and 50°C and analysed for f‐Hb concentration at days 0, 3, 7, 10, 14, 21, and 28. Relative f‐Hb (percentage change of day 0) in both buffer types was compared between temperatures at each timepoint. Generalised linear modelling identified variables influencing f‐Hb degradation.

**Results:**

SOC3 exhibited significant f‐Hb decline from day 10 at 23°C and from day 3 at all higher temperatures assessed (*p* < 0.05). In contrast, f‐Hb concentrations remained stable within SOC4 for up to 28 days at 23°C, with reductions observed after 7, 7, and 14 days at 50°C, 45°C, and 35°C, respectively (*p* < 0.05). Higher temperatures and longer storage duration significantly decreased f‐Hb concentration, while SOC4 demonstrated superior f‐Hb preservation relative to SOC3 (generalised linear model estimate = 15.4, *p* < 0.001).

**Conclusion:**

The SOC4 FIT buffer improves f‐Hb stability compared with the current formulation and may improve CRC screening accuracy, particularly in high‐temperature environments.

## Graphical abstract

This study investigated faecal haemoglobin (f‐Hb) stability in a new faecal immunochemical test (FIT) buffer formulation, where fresh stool was incubated in buffer under varying temperature and time conditions. Relative to the buffer formulation currently in use (OC‐Sensor SOC3), the new formulation (OC‐Sensor SOC4) could retain more f‐Hb at higher temperatures and for longer durations. This may improve FIT‐based population screening for colorectal cancer, particularly in hot climates and rural areas, where time to FIT‐analysis may be longer due to distance.

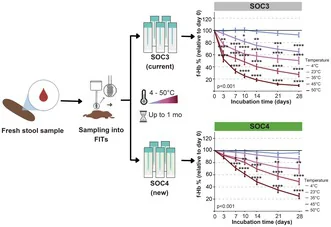

## 1 Background

Colorectal cancer (CRC) is the third most commonly diagnosed cancer and the second leading cause of cancer‐related death worldwide, with over 1.5 million new cases and 900,000 CRC‐related deaths in 2022 [1]. CRC has greater incidence in developed countries, likely due to lifestyle and environmental factors, and incidence in individuals under the age of 50 years is on the rise [2]. However, over recent decades overall CRC incidence and mortality rates have decreased, largely due to adoption of CRC screening [2, 3]. The main forms of CRC screening are endoscopic direct‐visualisation methods (e.g., flexible sigmoidoscopy or colonoscopy), and non‐invasive two‐step methods such as faecal occult blood tests (FOBT) followed by diagnostic endoscopic direct‐visualisation after a positive FOBT test [3]. Immunochemical FOBT (FIT) has replaced the use of guaiac faecal occult blood tests (gFOBT) due to improved sensitivity and methodology (FIT does not require the dietary and medication restrictions associated with gFOBT) [4]. However, the diagnostic accuracy of FIT for CRC and advanced adenoma detection can be influenced by pre‐analytical conditions [5], particularly ambient temperature [6, 7, 8].

FIT is now the main form of screening worldwide [9]. It quantifies the globin moiety of human haemoglobin (Hb) in faeces, with levels above a set positivity threshold (specified by each country or jurisdiction) indicating colorectal blood loss and the recommendation for diagnostic colonoscopy [10]. Test sensitivity for CRC can range from 25% to 100% with varying positivity thresholds [11]. The measured faecal Hb (f‐Hb) concentration decreases with increasing temperature [6, 7, 8, 12, 13, 14], due to the temperature‐induced f‐Hb degradation [7, 15]. Consequently, FIT positivity and sensitivity for detecting advanced neoplasia (CRC and advanced adenomas) also decrease [6, 7]. As the purpose of FIT‐based population screening is to detect CRC and pre‐cancerous lesions at an early stage [16], it is crucial that f‐Hb is effectively preserved from the time of sampling to the time it reaches the testing laboratory, regardless of ambient temperature.

Previous research suggests that only 62% of screening participants adhere to the recommended storage of samples at 4°C prior to their return to the laboratory, and that ambient temperature during sample transit as well as the time taken for return to the laboratory can vary substantially [8]. Therefore, it is important that sample collection devices contain buffer with adequate f‐Hb preservation properties. The OC‐Sensor FIT (EIKEN CHEMICAL CO. LTD, Tokyo, Japan) is used in over 20 countries for CRC screening [10]. Refinement of the OC‐Sensor FIT sample collection device buffer formulation over the years has improved f‐Hb preservation ability [17, 18]. In 2017 we showed that 80% of FIT f‐Hb content was preserved for up to 20 days at room temperature in a new OC‐Sensor FIT sample buffer formulation, compared to only 8 days in the previous formulation [17]. Additionally, the new buffer could preserve 80% of FIT f‐Hb for up to 8 days when samples were stored at 35°C [17]. Another study also observed improvement in FIT f‐Hb preservation, finding a 10% decrease in f‐Hb concentration at room temperature in the new buffer, compared to a 40% decrease for samples stored in the original buffer [18]. However, these improvements did not translate to a clinically relevant improvement to the detection of advanced neoplasia [18]. Additional enhancement of the FIT buffer may therefore be warranted—particularly to support CRC screening in regions experiencing extreme heat that are located long distances from the testing laboratory, such as Central Australia and parts of Western Australia.

To address this, a new formulation buffer has been developed for the OC‐Sensor FIT collection devices (‘SOC4’). This study compared the stability of Hb in faecal samples collected in the new SOC4 collection buffer to the current formulation that were incubated over varying temperatures and durations.

## 2 Methods

### 2.1 Overview

Laboratory experiments were conducted to compare the stability of f‐Hb in the new OC‐Sensor FIT sample buffer (SOC4) against the current buffer (SOC3) when exposed to increasing temperature and time. Fresh whole faecal samples were collected by participants with known colorectal blood loss and sampled into 15 SOC3 and 15 SOC4 FIT collection devices. Buffer solutions were then stored at set temperatures for up to 28 days. F‐Hb concentration relative to baseline was compared between the SOC4 and SOC3 buffers at set temperatures and timepoints.

### 2.2 Study Population

The investigations were part of a larger study (approved by Southern Adelaide Clinical Human Research Ethics Committee (reference number 35.20); and registered in Australia and New Zealand Clinical Trials registry (#12620000668909)). The study cohort was those scheduled for colonoscopy, and if the indication for the procedure was rectal bleeding or a positive FIT, individuals were invited to provide an additional faecal sample. Written informed consent was provided by participants.

### 2.3 Sample Collection, Preparation and Analysis

Participants were mailed a study package containing study information, a stool sample collection pot, gloves, and an ice pack. They were requested to collect a small faecal sample at home (roughly the size of a cherry) into the collection pot and place it in a bag with the ice pack. The participant returned the faecal sample on ice directly to the testing laboratory (Flinders Centre for Innovation in Cancer, Bedford Park, South Australia) within 24 h.

**FIGURE 1:**
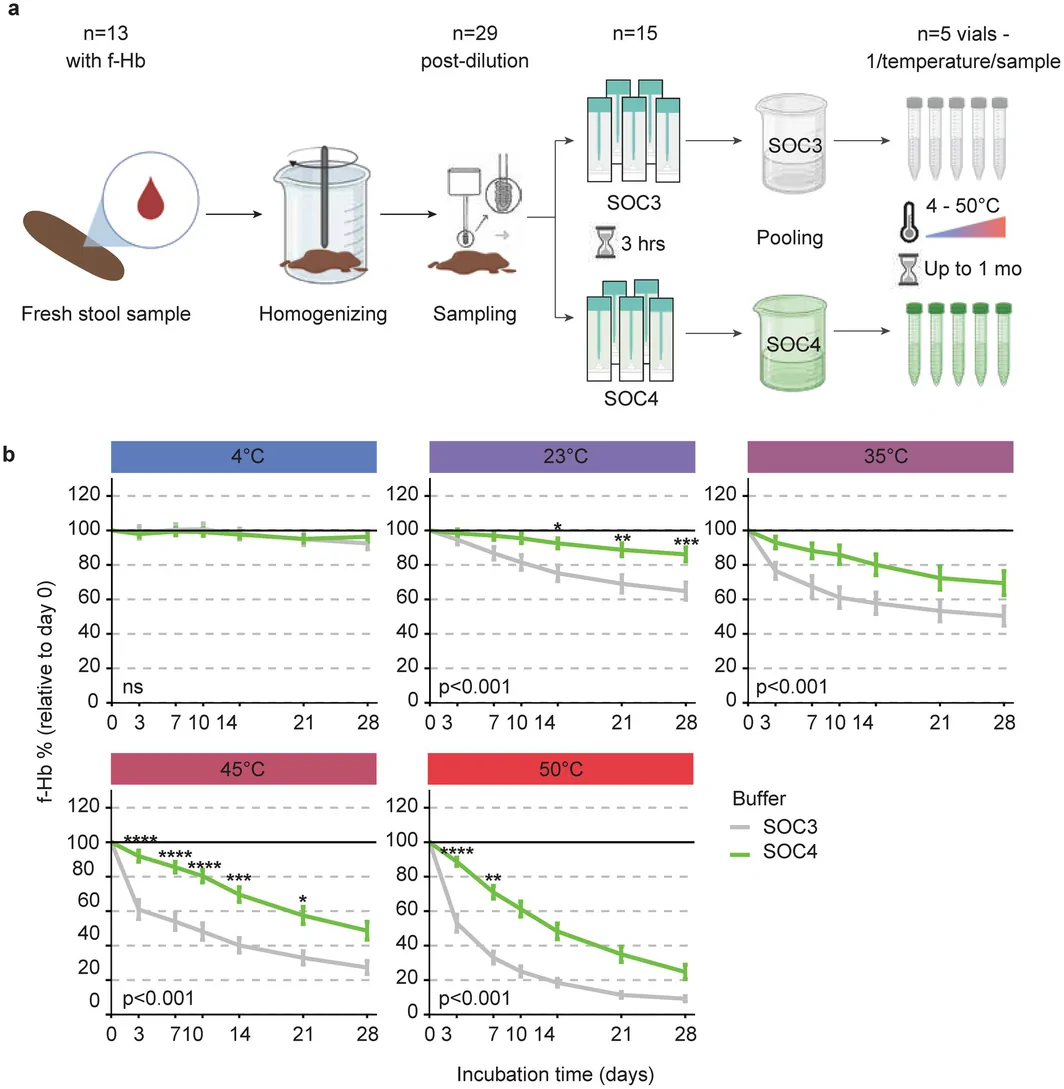
(a) Stool collection, preparation and analysis (*n* = 13 stool samples, *n* = 29 faecal‐buffer samples after dilution). (b) Relative change in faecal haemoglobin (f‐Hb) between SOC3 (grey) and SOC4 (green) buffers at temperatures over time (all concentrations, *n* = 29). Asterisks denote significant differences between SOC3 versus SOC4 at given timepoints. *p < 0.05, **p < 0.01, ***p < 0.001, ****p < 0.0001. *p*‐values denote interaction effect of buffer type on relative f‐Hb change.

The sample collection, preparation and analysis procedures are displayed in Figure 1a. At the laboratory, the faecal sample was mixed to create a more homogeneous sample, and a standard amount (~10 mg) was sampled into a single FIT collection device (OC‐Auto Sampling Bottle, EIKEN CHEMICAL CO. LTD). The device was analysed for FIT f‐Hb concentration with the OC‐Sensor Pledia Autoanalyser (EIKEN CHEMICAL CO. LTD) as per the manufacturer's instructions. Where the f‐Hb concentration was ≥ 45 μg Hb/g faeces, dilutions were created by mixing a portion of the sample with a fresh faecal sample from a donor that had no detectable f‐Hb, to create additional samples in the range of: 5–15, 24 (±20%), 36 (±20%), 48 (±20%), 96 (±20%), and > 120 μg Hb/g faeces. The faeces were then sampled into 15 FIT OC‐Auto Sampling Bottle 3 (SOC3) and 15 OC‐Auto Sampling Bottle 4 (SOC4). The FIT collection devices were incubated for 3 h at room temperature, after which the faecal‐buffer mixtures were extracted from the FIT collection device and pooled (separately for each buffer type) before being divided equally into 5 screw‐top vials. The faeces‐buffer mixtures were then centrifuged at 2000 g for 10 min to pellet the debris, and 300 μL of the supernatants were dispensed into separate sample cups and analysed with the OC‐Sensor Pledia Autoanalyser for time zero f‐Hb concentration in duplicate. Following analysis, the faecal‐buffer solutions were resuspended in the screw‐top vials, with the lids sealed with sealing film (DuraSeal laboratory stretch film, 3PI Supplies) to prevent sample loss. Samples were incubated at 4°C (refrigerator), 23°C (room temperature laboratory shelf), 35°C (standard laboratory incubator), 45°C (standard laboratory incubator) or 50°C (standard laboratory incubator) for 28 days. Temperature loggers were used throughout the testing period to ensure the temperatures were achieved and maintained. The faecal‐buffer solutions from each temperature group were analysed for f‐Hb following incubation at 3, 7, 10, 14, 21 and 28 days. Prior to f‐Hb assessment at each time point, samples were centrifuged (in the screw‐top vials) at 2000 *g* for 10 min, and a 300 μL aliquot of the supernatant was assayed as described for time zero samples. The remainder of the solution was resuspended in the screw‐top vials, sealed and returned to the original incubation conditions for future f‐Hb assessment at the remaining time points.

### 2.4 Statistical Analysis

F‐Hb concentrations were expressed as μg Hb/g faeces and percentage of day 0 concentrations (mean ± standard error of the mean (SEM)). Relative f‐Hb levels of faecal samples collected in the SOC3 and SOC4 buffers were compared between temperatures (4°C reference) and time points (day 0 reference) using ANOVA with a post hoc Tukey test for multiple comparisons. Generalised linear modelling was used to identify whether buffer formulation, starting f‐Hb concentration (with 24% ± 20% μg Hb/g selected as the reference, as 20 μg Hb/g faeces is the most commonly used positivity threshold in organised screening programmes) [10], incubation temperature and incubation time were associated with changes in f‐Hb levels. Sample summary statistics were presented as median, interquartile range (IQR) and range (minimum–maximum). Statistical analysis was conducted using RStudio software (Posit Software, Boston, MA). Results were deemed statistically significant at a *p* value < 0.05.

## 3 Results

Of the 27 individuals enrolled with known colorectal bleeding, 13 had adequate f‐Hb concentration in fresh stool to conduct the experiment (median age 55.8y, range: 30.8–71.4y; 7 female). Eight of the samples had f‐Hb ≥ 45 μg Hb/g faeces and were further diluted. These were combined with donor faecal samples with no detectable f‐Hb, to create additional baseline concentrations (lower than the original f‐Hb concentration). Overall, a total of 29 unique faeces‐buffer samples were created and analysed (Table 1).

**TABLE 1:** Summary statistics for included faecal samples. Starting faecal haemoglobin (f‐Hb) concentration was expressed as the average concentration of f‐Hb in SOC3 and SOC4 buffer on Day 0.

Initial f‐Hb concentration group	*N* (% total)	f‐Hb concentration (μg Hb/g faeces)		
		Median	Interquartile range	Range (Min – Max)
All samples	29 (100)	41.0	23.8–93.6	5.6–160.0
~5–15 μg Hb/g faeces	5 (17.2)	8.6	8.4–10.4	5.6–13.8
24 μg Hb/g faeces (±20%)	4 (13.8)	23.7	23.2–23.9	22.2–24.2
36 μg Hb/g faeces (±20%)	6^a^ (20.7)	35.8	34.6–36.4	33.4–41.0
48 μg Hb/g faeces (±20%)	6^a^ (20.7)	47.0	45.6–48.4	41.0–57.6
64 μg Hb/g faeces^b^	1 (3.4)	64.0	N/A	N/A
96 μg Hb/g faeces (±20%)	5 (17.2)	96.2	95.63–98.6	93.6–111.6
> 120 μg Hb/g faeces	3 (10.3)	158.8	148.3–159.4	137.8–160.0

^a^ One sample overlaps 36 and 48 μg Hb/g groups, due to ±20% allowance in concentration value.

^b^ was not analysed as a concentration group due to small sample size but was included in the analyses of all samples.

Table 1 displays the 6 starting concentration ranges that were considered. One sample which had a starting concentration of 41 μg Hb/g faeces was considered in both the 36 μg Hb/g faeces and 48 μg Hb/g faeces f‐Hb groups. One sample with a starting concentration of 64 μg Hb/g faeces was not captured within any of the concentration groups. This sample was excluded from analyses of individual concentration groups, but was included when samples of all concentrations combined were analysed.

A direct comparison of the performance of the SOC3 against SOC4 buffer in preserving f‐Hb at different temperatures over time is displayed in Figure 1b. Compared to the SOC3 buffer, the SOC4 buffer preserved significantly more f‐Hb for up to a week at temperatures ≥ 45°C (*p* < 0.05) and from day 14 onwards at 23°C (*p* < 0.05) (Figure 1b), when results from all samples were grouped together irrespective of the starting f‐Hb concentration. In samples with a day 0 f‐Hb concentration of 36 μg Hb/g faeces, the SOC4 buffer had significantly better f‐Hb preservation on days 14–21 when stored at 45°C (*p* < 0.05) and on days 3–21 when stored at 50°C (*p* < 0.01) (Figure S1c). In samples with a day 0 f‐Hb concentration of 48 and 96 μg Hb/g faeces, SOC4 buffer retained significantly more f‐Hb at all timepoints when stored at 45°C and 50°C (*p* < 0.05) (apart from the 96 μg Hb/g faeces samples stored at 50°C on day 28; Figure S1d,e). In samples with a starting f‐Hb concentration of > 120 μg Hb/g faeces, significantly greater f‐Hb retention was observed in SOC4 buffer relative to SOC3 on days 7–10 when stored at 35°C (*p* < 0.05), on days 3–21 when stored at 45°C (*p* < 0.05), and on days 3–7 when stored at 50°C (*p* < 0.01) (Figure S1f). Both SOC3 and SOC4 had comparable f‐Hb preservation in samples with a starting concentration of ~5–15 and 24 μg Hb/g faeces (Figure S1a,b), keeping in mind that due to low starting concentrations, small fluctuations in f‐Hb lead to larger percentage changes. A direct comparison of the performance of SOC3 against SOC4 buffer (with all samples grouped together regardless of the starting f‐Hb concentration) is also displayed at different time points in Figure S2.

**FIGURE 2:**
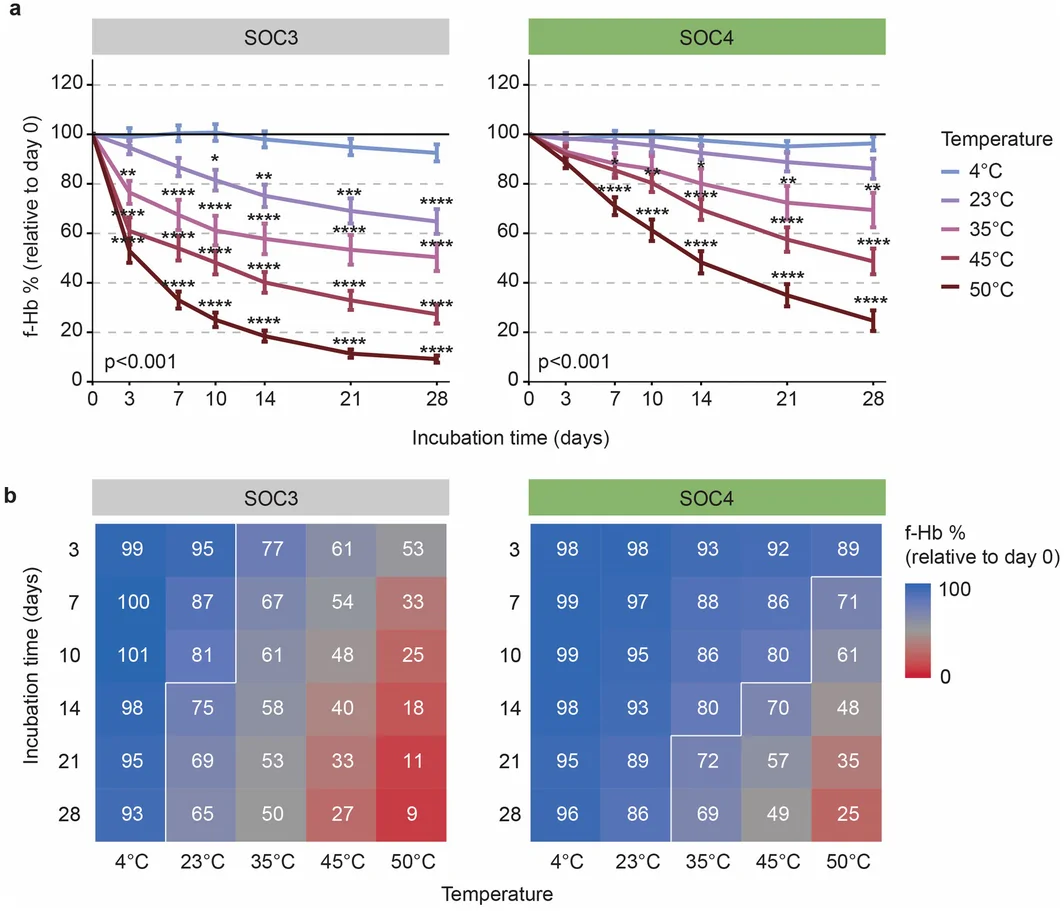
(a) Relative change in faecal haemoglobin (f‐Hb) in SOC3 and SOC4 buffers (all concentrations, *n* = 29). Asterisks indicate significant differences (compared with temperature 4°C) at given timepoints. *p < 0.05, **p < 0.01, ***p < 0.001, ****p < 0.0001. *p*‐values denote interaction effect of temperature. (b) Heatmap of f‐Hb relative change in SOC3 and SOC4 buffers (all concentrations, *n* = 29). White line divides preservation of ≥ 80% of starting f‐Hb concentration.

When results from all samples were grouped together for analysis (irrespective of starting f‐Hb concentration), those incubated at ≥ 23°C in SOC3 buffer and ≥ 35°C in SOC4 had a significant reduction in f‐Hb over time, with increasing temperatures causing a greater decline (Figure 2a). At the highest incubation temperature (50°C) and longest duration (28 days), f‐Hb decreased to 9.2% of the starting f‐Hb concentration in the SOC3 buffer (*p* < 0.0001), while 24.7% of the starting concentration was preserved in the SOC4 buffer (*p* < 0.0001, Figure 2a). The first significant reductions in f‐Hb concentration were observed after 3 days of storage at ≥ 35°C (versus 4°C) in SOC3 buffer (*p* < 0.01), whereas in SOC4 buffer, a significant reduction in f‐Hb was observed at day 7 at a storage temperature of 45°C (*p* < 0.05, Figure 2a).

Changes in f‐Hb levels at various starting f‐Hb concentrations were also assessed (Figure S3). For the groups where f‐Hb concentrations were within ranges that are commonly used in organised screening programmes (between approximately 20 to 47 μg Hb/g faeces) [10], the decline in f‐Hb within SOC3 buffer varied depending on the starting f‐Hb concentration. For 24 μg Hb/g faeces, a significant reduction in f‐Hb was observed from day 7 at 50°C (*p* < 0.05) and at day 28 at 45°C (*p* < 0.05, Figure S3b). For the starting concentration of 48 μg Hb/g faeces, f‐Hb was significantly reduced as early as day 3 at ≥ 35°C (*p* < 0.01), and significantly declined from day 10 at 23°C (*p* < 0.05, Figure S3d). For the SOC4 buffer, at the starting concentration of 24 μg Hb/g faeces, a significant decrease in f‐Hb was only observed from 14 days onwards at 50°C (*p* < 0.05, Figure S3b); while at 48 μg Hb/g faeces, the reduction was only observed at 28 days at 50°C (*p* < 0.05, Figure S3d).

Additionally, the reductions in f‐Hb in SOC3 and SOC4 expressed as a percentage of day 0 are presented as a heatmap in Figure 2b, with 80% f‐Hb preservation being selected as a threshold for comparison as per our previous study [17]. When considering all concentrations, the SOC4 buffer had a longer retention of ≥ 80% f‐Hb content relative to starting concentrations, over longer time periods and higher temperatures compared to SOC3 buffer. This pattern was observed when samples were grouped by starting f‐Hb concentration (Figure S4), apart from samples with a starting f‐Hb concentration of 24 μg Hb/g faeces.

Generalised linear modelling was conducted to identify factors affecting the change in f‐Hb from day 0 (Table 2). SOC4 buffer was associated with a significantly higher retention of f‐Hb concentration in comparison to the SOC3 buffer (estimate = 15.8, *p* < 0.001). Increasing sample incubation temperature was associated with a significantly greater f‐Hb concentration loss (estimate = −10.8, *p* < 0.001 at 23°C; estimate = −48.8, *p* < 0.001 at 50°C), as was increasing incubation time (estimate = −15.5, *p* < 0.001 at day 3; estimate = −43.5, *p* < 0.001 at day 28). Relative to 24 μg Hb/g faeces, samples with higher starting f‐Hb concentrations were associated with a significantly greater decrease in f‐Hb levels (estimate = −4.7, *p* = < 0.01 for 36 μg Hb/g; estimate = −14.4, *p* < 0.001 for > 120 μg Hb/g).

**TABLE 2:** Factors affecting faecal haemoglobin (f‐Hb) changes from day 0 concentrations.

	Estimate^a^	Standard error	*p*
**Buffer**			
SOC3	0.0		Reference
SOC4	15.8	1.0	< 0.001
**Starting f‐Hb concentration**			
~5–15 μg/g faeces	−1.3	1.9	0.492
24 μg/g faeces	0.0		Reference
36 μg/g faeces	−4.7	1.8	< 0.01
48 μg/g faeces	−2.3	1.8	0.201
96 μg/g faeces	−4.7	1.8	< 0.05
> 120 μg/g faeces	−14.4	2.1	< 0.001
**Temperature**			
4°C	0.0		Reference
23°C	−10.8	1.6	< 0.001
35°C	−22.9	1.6	< 0.001
45°C	−33.5	1.6	< 0.001
50°C	−48.8	1.6	< 0.001
**Time**			
0 days	0.0		Reference
3 days	−15.5	1.9	< 0.001
7 days	−22.1	1.9	< 0.001
10 days	−26.6	1.9	< 0.001
14 days	−32.4	1.9	< 0.001
21 days	−39.3	1.9	< 0.001
28 days	−43.5	1.9	< 0.001

^a^ Analysed with generalised linear modelling.

## 4 Discussion

This study investigated the effect of incubation temperature and time on faecal haemoglobin stability in a newly formulated OC‐Sensor FIT sample buffer in vitro. We show that the new buffer improved preservation of f‐Hb at higher temperatures and for longer incubation times over the currently used formulation. When samples were stored at room temperature (23°C), there was a significant decrease in f‐Hb concentration starting at 7 days in the current buffer formulation, whereas no significant decrease was observed during 28 days with the new formulation. When compared directly, the new buffer preserved a significantly greater amount of f‐Hb up to 7 days when samples were exposed to more extreme temperatures (45°C and 50°C) compared to the current formulation. The new formulation was also able to retain more than 80% of the starting f‐Hb concentration for a greater range of temperatures and durations. These data provide evidence to support at home FIT‐based CRC screening programmes in rural/regional areas that experience extreme heat.

In line with current literature [17, 19], our results confirm refrigeration is the most suitable storage option to maximise f‐Hb preservation, and we show this regardless of the sample buffer formulation. However, approximately 1 in 3 patients do not refrigerate their samples prior to return [8]. Considering the low rate of patient compliance with sample refrigeration, it is likely that FIT samples will be exposed to higher temperatures during transit. With variable times for transit [20], it is therefore important that the FIT sample buffer can effectively preserve haemoglobin at higher temperatures and for long periods of time.

The newly formulated SOC4 buffer investigated may be able to better preserve f‐Hb compared to other buffers on the market. A previous study found that the NS Plus FIT (Alfresa Pharma, Osaka, Japan) could preserve 80% of the f‐Hb for less than 7 days at room temperature [21], whereas the new OC‐Sensor SOC4 buffer could preserve over 80% for up to 28 days. At 45°C, the NS Plus FIT preserved less than 60% of Hb over 7 days [21], whereas the new SOC4 buffer preserved over 80% for the same duration. The FOB‐Gold FIT (Sentinel Diagnostics, Milano, Italy) preserved 80% of f‐Hb from FIT positive patients for 7 days when incubated at 30°C [15], whereas the OC‐Sensor SOC4 buffer could preserve 88% for the same duration at 35°C. However, it must be noted that sample conditions varied between studies. The NS Plus FIT used fresh faecal samples spiked with set quantities of haemolysed whole blood [21], and the FOB‐Gold FIT used patient samples collected up to 1 week before the laboratory experiments commenced [15]. Our study used fresh faecal samples from individuals with known colorectal bleeding, processed almost immediately in the laboratory. Caution should be taken when interpreting studies that have used faecal samples spiked with haemolysed whole blood; there may be differences in Hb binding within the faecal matrix and age of blood within natural samples, where Hb is less stable [22].

Effective f‐Hb preservation in FIT samples is crucial to ensure that the sensitivity of screening for advanced neoplasia is not compromised [6]. Variables that are unique to different screening programmes which can reduce FIT Hb levels, and therefore sensitivity, include the climate of the screening region, brand of FIT utilised, and FIT positivity thresholds [5]. For example, a study conducted in California found that sensitivity for advanced neoplasia was significantly lower in summer (75.0%) compared to winter (76.4%) [6]. Other studies in South Korea and Spain found that FIT positivity decreased at higher temperatures, but not sensitivity for advanced neoplasia [23, 24]. The strong heat‐stability of our SOC4 buffer suggests suitability for use in a wide variety of climates and the potential for maintaining sensitivity for advanced neoplasia, although this needs to be tested in a clinical setting. It is notable that the SOC4 buffer had improved f‐Hb preservation relative to SOC3 in patient faecal samples with haemoglobin concentrations starting at 24 μg/g faeces, which is similar to the 20 μg/g threshold used in many screening programmes [10]. FIT positivity thresholds used in screening programmes vary across the world [10]. The new SOC4 buffer would likely maintain sensitivity for advanced neoplasia over many of these different positivity thresholds, given its improved f‐Hb preservation capacity across multiple concentrations. This may be particularly useful in countries that use higher positivity thresholds [10], as we showed that samples with higher starting f‐Hb concentrations degraded at a faster rate than those with lower f‐Hb levels, and others have found that sensitivity for advanced neoplasia decreases with higher temperatures when higher positivity thresholds are applied [7].

A strength of this study was the use of fresh faecal samples with endogenously elevated f‐Hb due to colorectal bleeding, rather than synthetic Hb/red cell lysate‐spiked faecal samples, as it more accurately represents a true clinical scenario. Additionally, most studies investigating temperature‐related Hb degradation in FIT collection devices were conducted more than 5 years ago to the best of our knowledge, highlighting the need for this study. A limitation of this study was the small number of unique samples, where only approximately half of enrolled participants had a testable level of Hb. Degradation of the f‐Hb could have already occurred due to the time taken for transporting the sample into the laboratory, resulting in less eligible samples.

Overall, the new formulation of the OC‐Sensor FIT SOC4 buffer has superior haemoglobin‐stabilising ability compared to the current buffer formulation used in the OC‐Sensor FIT. This may improve CRC screening programme sensitivity for advanced neoplasia, especially in regional/rural areas and hot climates. Transition to the new SOC4 buffer could therefore improve equity of screening programmes across communities that currently lack access to CRC screening due to time‐ and temperature‐dependent restrictions. Future research should now evaluate whether the new OC‐Sensor FIT buffer can improve the sensitivity for detection of advanced colorectal neoplasia in a CRC screening or clinical cohort.

## Author Contributions

R.R.: data curation, investigation, methodology, writing – original draft, writing – review and editing; G.L.‐L.: conceptualisation, data curation, formal analysis, investigation, methodology, project administration, visualisation, writing – review and editing; J.M.W.: funding acquisition, formal analysis, visualisation, supervision, writing – review and editing; K.W.: investigation, writing – review and editing; R.P.: project administration, resources, writing – review and editing; G.P.Y.: conceptualisation, funding acquisition, methodology, writing – review and editing; E.L.S.: conceptualisation, data curation, funding acquisition, investigation, methodology, project administration, supervision, writing – review and editing.

## Funding

Consumables, equipment, reagents and funding were received from EIKEN CHEMICAL CO. LTD.

## Ethics Statement

The study was approved by the Southern Adelaide Clinical Human Research Ethics Committee.

## Consent

Written informed consent was obtained by participants prior to participation.

## Conflicts of Interest

The study received institutional support from EIKEN CHEMICAL CO. LTD, which had no involvement in data analysis or reporting. E.L.S. is an unpaid member of the International Federation for Clinical Chemistry and Laboratory Medicine faecal immunochemical test (FIT) working group. G.P.Y. is an advisor to Health‐First Systems in the design of a novel FIT.

## Supporting information


**Figure S1:** Relative change in faecal haemoglobin (f‐Hb) between SOC3 (grey) and SOC4 (green) buffers across temperatures and time. (a) 5–15 μg Hb/g faeces (*n* = 5) (b) 24 μg/g (*n* = 4) (c) 36 μg/g (*n* = 6) (d) 48 μg/g (*n* = 6) (e) 96 μg/g (*n* = 5) (f) > 120 μg/g (*n* = 3). Asterisks indicate significant SOC3–SOC4 differences at given timepoint. *p < 0.05, **p < 0.01, ***p < 0.001.
**Figure S2:** Relative change in faecal haemoglobin (f‐Hb) between SOC3 (grey) and SOC4 (green) buffers at different temperatures over time (all concentrations, *n* = 29). Asterisks indicate significant SOC3‐SOC4 differences. *p < 0.05, **p < 0.01, ***p < 0.001.
**Figure S3:** Relative change in faecal haemoglobin (f‐Hb) in SOC3 and SOC4 buffers: (a) 5–15 μg Hb/g (*n* = 5) (b) 24 μg/g (*n* = 4) (c) 36 μg/g (*n* = 6) (d) 48 μg/g (*n* = 6) (e) 96 μg/g (*n* = 5) (f) > 120 μg/g (*n* = 3). Asterisks indicate significant differences at a given time compared with temperature 4°C. Data are mean ± standard error of mean. *p < 0.05, **p < 0.01, ***p < 0.001.
**Figure S4:** Heatmap showing mean faecal haemoglobin (f‐Hb) concentration (% day 0) for samples in SOC3 and SOC4 buffers. (a) ~5–15 μg Hb/g faeces (*n* = 5) (b) 24 μg/g (*n* = 4) (c) 36 μg/g (*n* = 6) (d) 48 μg/g (*n* = 6) (e) 96 μg/g (*n* = 5) (f) > 120 μg Hb/g (*n* = 3). White line divides timepoints and temperatures where ≥ 80% of starting concentration was preserved.

## Data Availability

The data that support the findings of this study are available from the corresponding author upon reasonable request.
