# Supplementary material for: A New Faecal Immunochemical Test Buffer With Improved Haemoglobin Stability Under Varying Temperature and Storage Times
Source: J Clin Lab Anal. 2026 Jul 26:e70317. Online ahead of print. doi: 10.1002/jcla.70317 (PMC13401936; doi:10.1002/jcla.70317)
Supplement: Supplementary file 1 — Figure S1: Relative change in faecal haemoglobin (f‐Hb) between SOC3 (grey) and SOC4 (green) buffers across temperatures and time. (a) 5–15 μg Hb/g faeces (n = 5) (b) 24 μg/g (n = 4) (c) 36 μg/g (n = 6) (d) 48 μg/g (n = 6) (e) 96 μg/g (n = 5) (f) > 120 μg/g (n = 3). Asterisks indicate significant SOC3–SOC4 differences at given timepoint. *p < 0.05, **p < 0.01, ***p < 0.001. Figure S2: Relative change in faecal haemoglobin (f‐Hb) between SOC3 (grey) and SOC4 (green) buffers at different temperatures over time (all concentrations, n = 29). Asterisks indicate significant SOC3‐SOC4 differences. *p < 0.05, **p < 0.01, ***p < 0.001. Figure S3: Relative change in faecal haemoglobin (f‐Hb) in SOC3 and SOC4 buffers: (a) 5–15 μg Hb/g (n = 5) (b) 24 μg/g (n = 4) (c) 36 μg/g (n = 6) (d) 48 μg/g (n = 6) (e) 96 μg/g (n = 5) (f) > 120 μg/g (n = 3). Asterisks indicate significant differences at a given time compared with temperature 4°C. Data are mean ± standard error of mean. *p < 0.05, **p < 0.01, ***p < 0.001. Figure S4: Heatmap showing mean faecal haemoglobin (f‐Hb) concentration (% day 0) for samples in SOC3 and SOC4 buffers. (a) ~5–15 μg Hb/g faeces (n = 5) (b) 24 μg/g (n = 4) (c) 36 μg/g (n = 6) (d) 48 μg/g (n = 6) (e) 96 μg/g (n = 5) (f) > 120 μg Hb/g (n = 3). White line divides timepoints and temperatures where ≥ 80% of starting concentration was preserved. [file JCLA-9999-e70317-s001.docx]

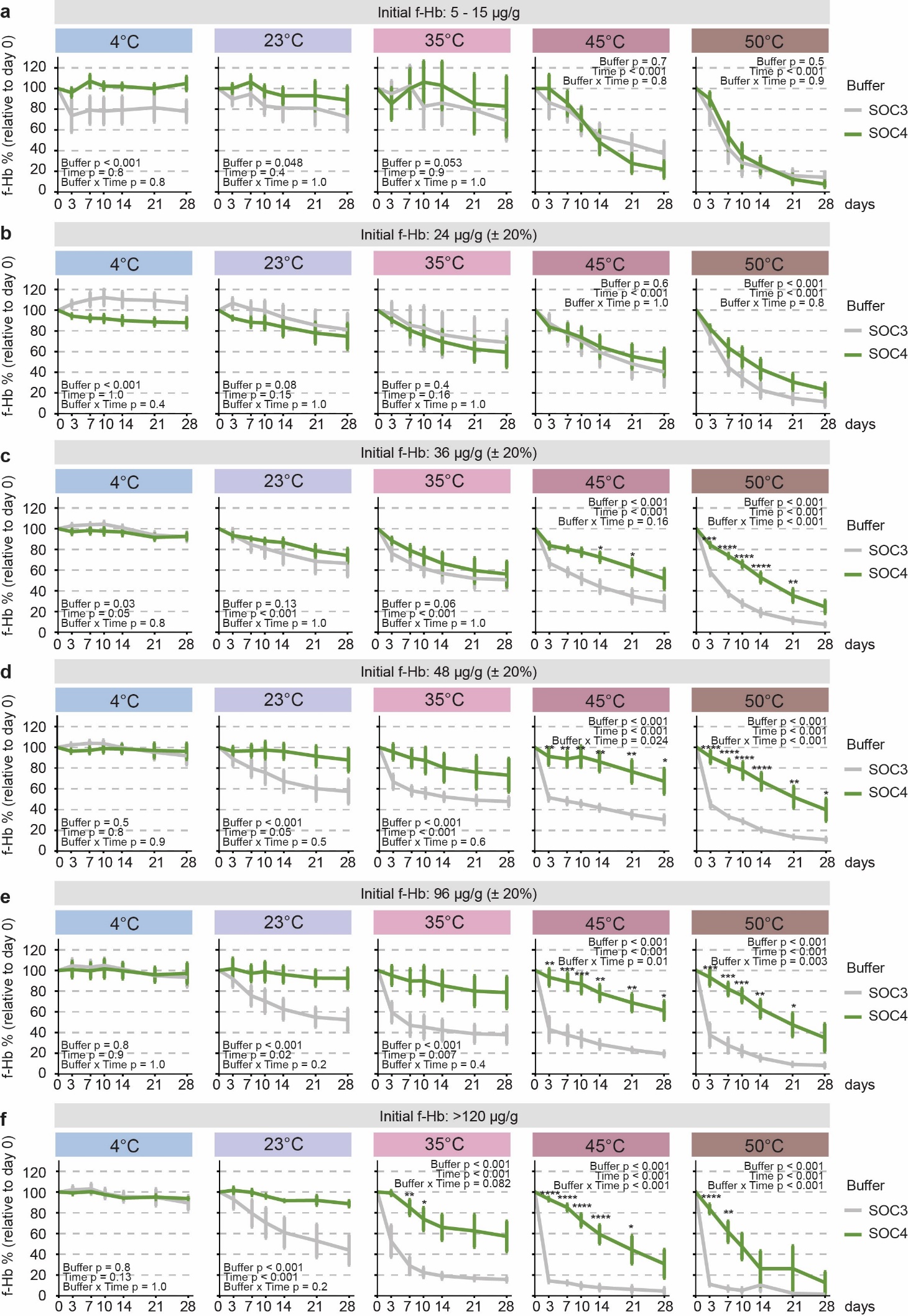


**Supplementary Figure 1.** Relative change in faecal haemoglobin (f-Hb) between

SOC3 (grey) and SOC4 (green) buffers across temperatures and time. **a)** 5–15 μg

Hb/g faeces (n=5) **b)** 24 μg/g (n=4) **c)** 36 μg/g (n=6) **d)** 48 μg/g (n=6) **e)** 96 μg/g (n=5)

**f)** >120 μg/g (n=3). Asterisks indicate significant SOC3–SOC4 differences at given

timepoint. **p*<0.05, ***p*<0.01, ****p*<0.001.


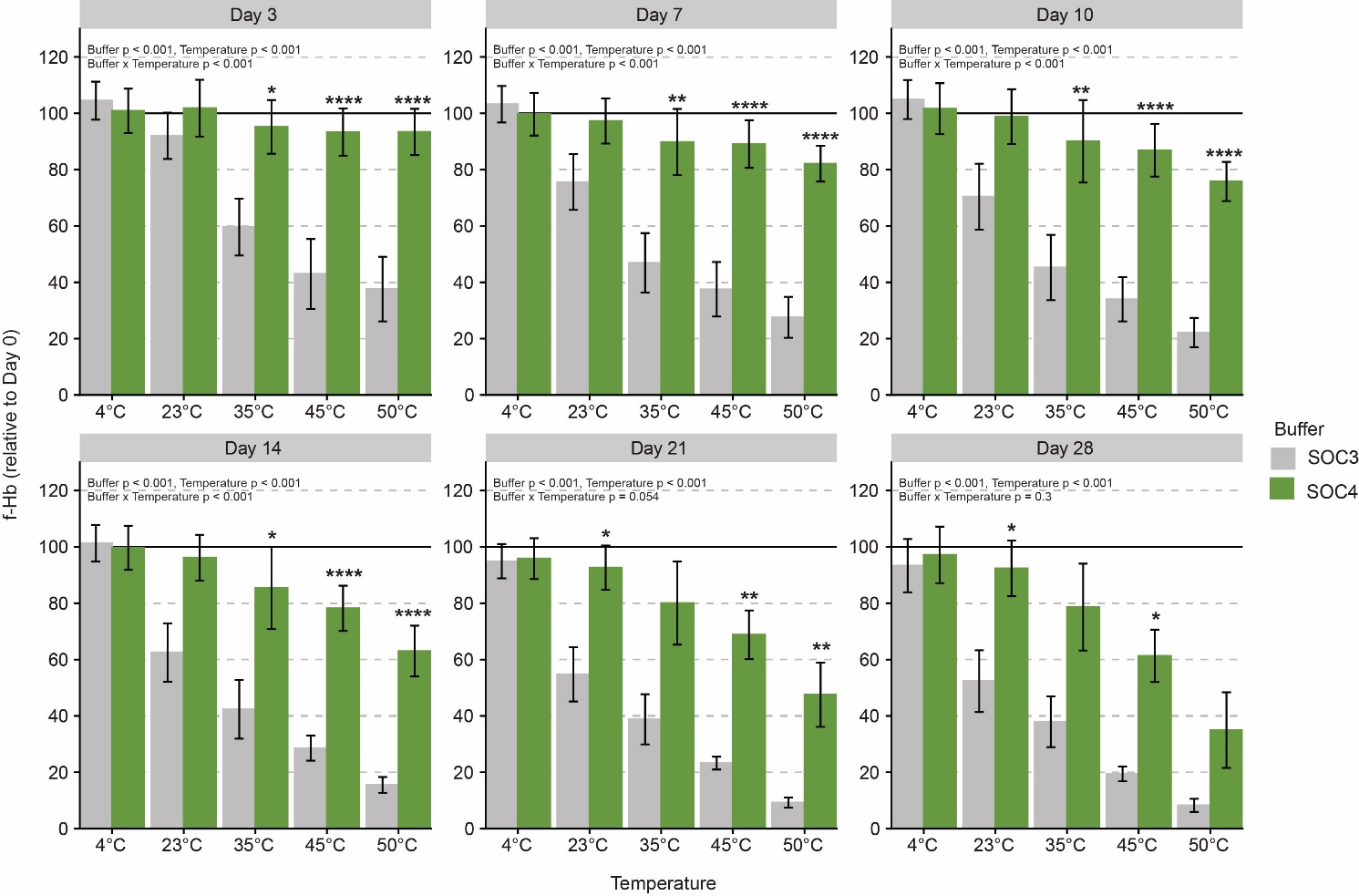


**Supplementary Figure 2**. Relative change in faecal haemoglobin (f-Hb) between

SOC3 (grey) and SOC4 (green) buffers at different temperatures over time (all

concentrations, n=29). Asterisks indicate significant SOC3-SOC4 differences.

**p*<0.05, ***p*<0.01, ****p*<0.001.


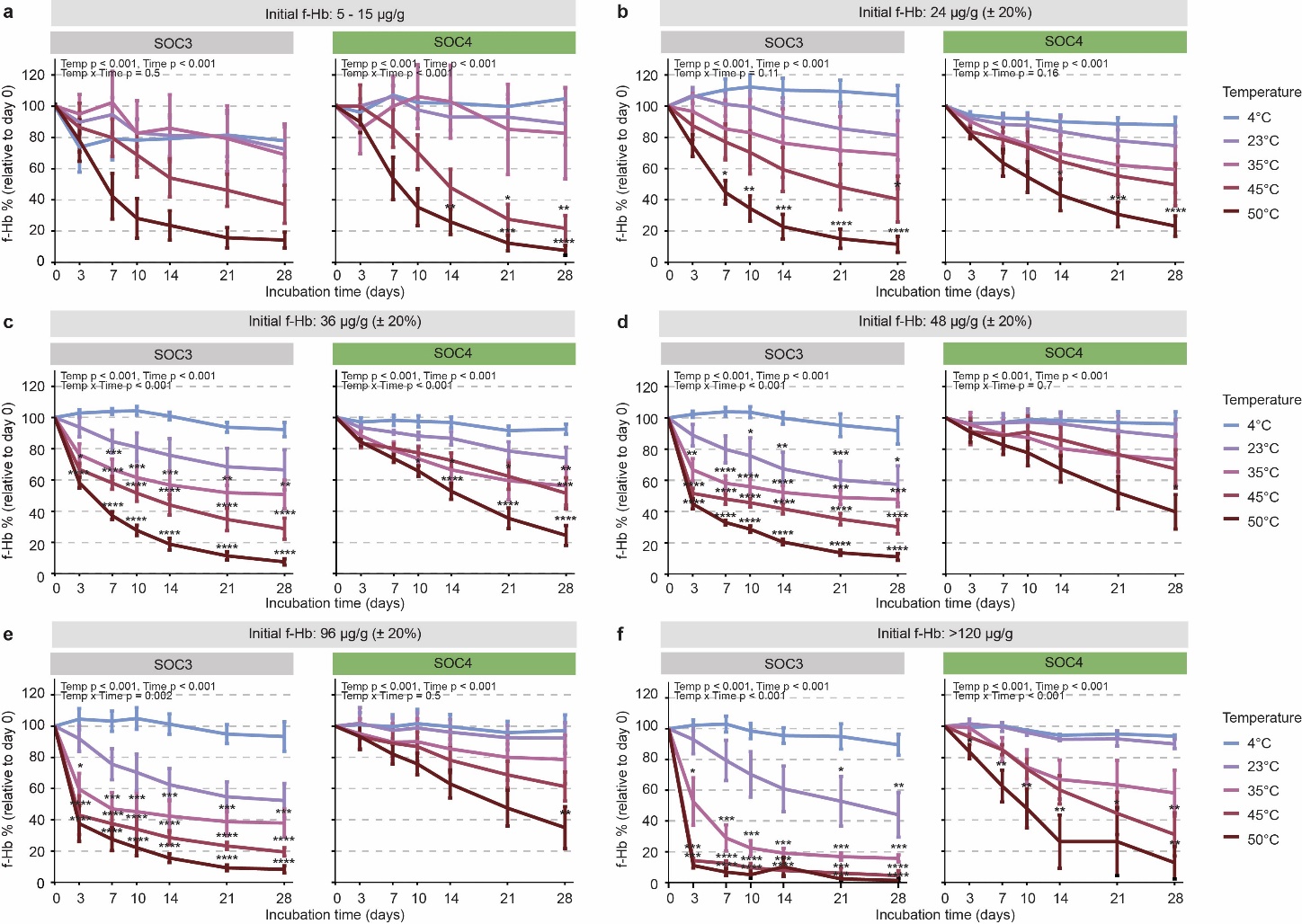


**Supplementary Figure 3.** Relative change in faecal haemoglobin (f-Hb) in SOC3 and

SOC4 buffers: **a)** 5–15 μg Hb/g (n=5) **b)** 24 μg/g (n=4) **c)** 36 μg/g (n=6) **d)** 48 μg/g

(n=6) **e)** 96 μg/g (n=5) **f)** >120 μg/g (n=3). Asterisks indicate significant differences at

a given time compared with temperature 4°C. Data are mean ± standard error of mean.

**p*<0.05, ***p*<0.01, ****p*<0.001.


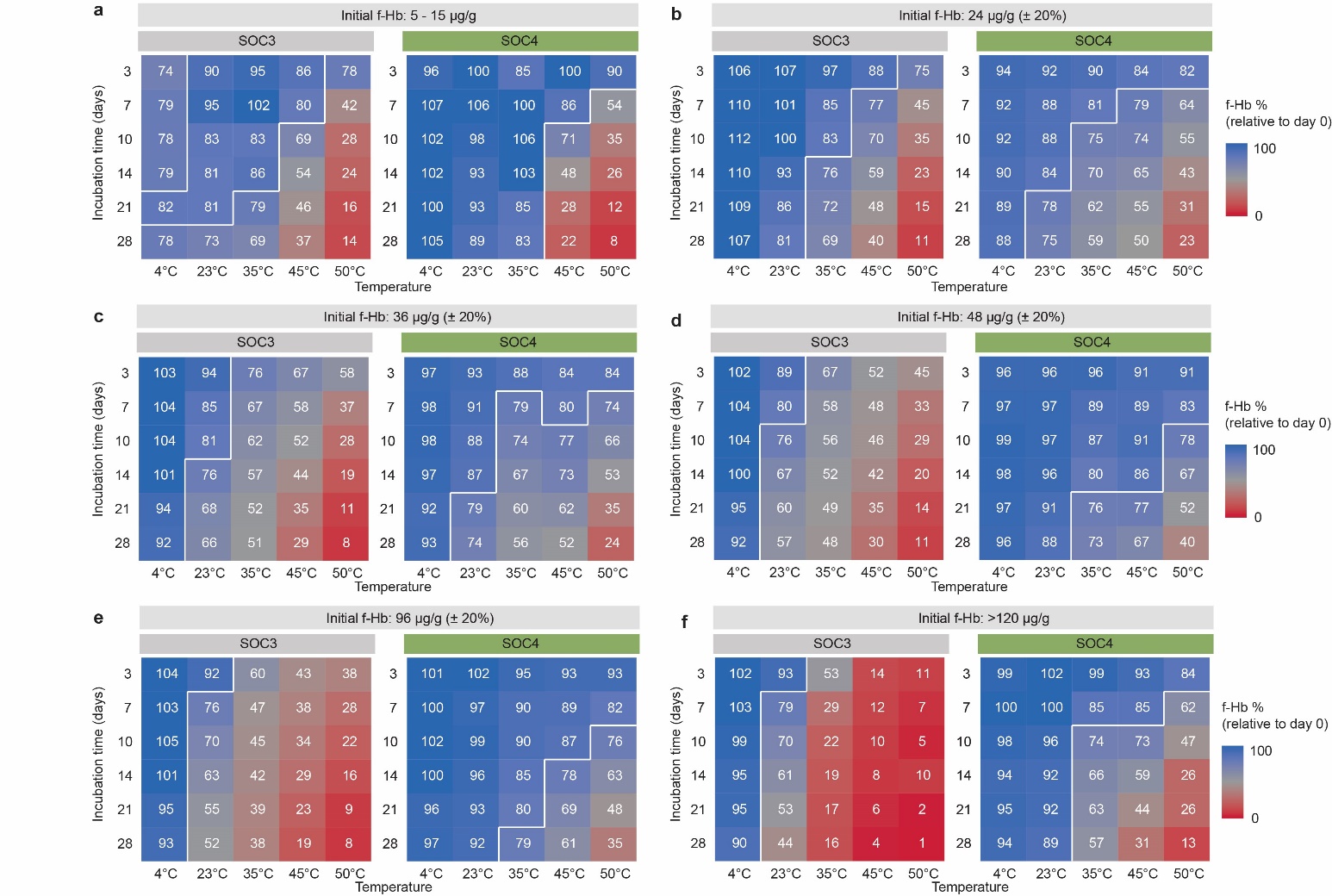


**Supplementary Figure 4.** Heatmap showing mean faecal haemoglobin (f-Hb)

concentration (% day 0) for samples in SOC3 and SOC4 buffers. **a)** ~5-15 μg Hb/g

faeces (n=5) **b)** 24 μg/g (n=4) **c)** 36 μg/g (n=6) **d)** 48 μg/g (n=6) **e)** 96 μg/g (n=5) **f)**

>120 μg Hb/g (n=3). White line divides timepoints and temperatures where ≥80% of

starting concentration was preserved.
